# CULTIVATING BELONGINGNESS: IN TEMPORARY SUPPORTIVE HOUSING FOR OLDER MALE VETERANS IN CALGARY, CANADA

**DOI:** 10.1093/geroni/igae098.1110

**Published:** 2024-12-31

**Authors:** Christine Walsh

**Affiliations:** University of Calgary, Calgary, Alberta, Canada

## Abstract

Veterans are two to three times more likely to experience homelessness compared to the general population, with an estimated 2,400 – 10,000+ homeless veterans in Canada. Veterans tend to be older, male, and more likely to cite an illness or medical condition as a contributing factor to their homelessness, than their non-veteran counterparts and thus have distinctly different needs. As part of the Aging in the Right Place (AIRP) partnership, a tri-city multi-methods study, we sought to understand the housing and support needs of older male military veterans (age 50+) living in a congregate temporary supportive housing program (a tiny home barracks), identified as a promising practice, in Calgary, Canada. In this presentation we draw on in-depth qualitative key informant interviews with service providers (n=5) and photovoice interviews with older shelter residents (n=10) to understand the shelter needs of older homeless veterans. Interviews were transcribed, managed with NVivo 1.6.1, and team-based flexible coding was employed on the interviews to determine older male veterans’ shelter needs. Thematic analysis of service provider interviews identified key features to promote AIRP included: program specific challenges and strengths and contexts, while eligibility and systemic barriers were noted as impediments to AIRP. Residents highlighted: autonomy and belonging, community and connection, ageism, routine and daily rhythm, safety, recognition, and the built environment as key to their (in)ability to AIRP in this promising practice. In this presentation we contextualize the study findings and offer recommendations for shelter and services designed to assist older housing insecure veterans to AIRP.

